# Regulation of Translation in the Protozoan Parasite *Leishmania*

**DOI:** 10.3390/ijms21082981

**Published:** 2020-04-23

**Authors:** Zemfira N. Karamysheva, Sneider Alexander Gutierrez Guarnizo, Andrey L. Karamyshev

**Affiliations:** 1 Department of Biological Sciences, Texas Tech University, Lubbock, TX 79409, USA; 2 Department of Cell Biology and Biochemistry, Texas Tech University Health Sciences Center, Lubbock, TX 79430, USA; sguarniz@ttuhsc.edu; 3 Programa de Estudio y Control de Enfermedades Tropicales, Universidad de Antioquia, Medellín 050010, Colombia

**Keywords:** *Leishmania* parasites, unicellular protozoa, parasite differentiation, translational control, selective translation, RNA-binding proteins, drug resistance, translational reprogramming, protein synthesis, ribosome

## Abstract

Leishmaniasis represents a serious health problem worldwide and drug resistance is a growing concern. *Leishmania* parasites use unusual mechanisms to control their gene expression. In contrast to many other species, they do not have transcriptional regulation. The lack of transcriptional control is mainly compensated by post-transcriptional mechanisms, including tight translational control and regulation of mRNA stability/translatability by RNA-binding proteins. Modulation of translation plays a major role in parasite survival and adaptation to dramatically different environments during change of host; however, our knowledge of fine molecular mechanisms of translation in *Leishmania* remains limited. Here, we review the current progress in our understanding of how changes in the translational machinery promote parasite differentiation during transmission from a sand fly to a mammalian host, and discuss how translational reprogramming can contribute to the development of drug resistance.

## 1. Introduction

*Leishmania* species are unicellular protozoans that cause leishmaniases affecting around 12 million people worldwide [1]. Currently 53 *Leishmania* species are recognized, with 20 of them pathogenic to humans [2]. Visceral leishmaniasis, also known as kala azar, is the most severe form of the disease with a mortality rate of almost 100% if untreated [3,4]. It is usually caused by *L. donovani* and *L. infantum*. The patients display enlargement of the spleen and liver, fever, and weight loss. Another type, mucocutaneous leishmaniasis, produces lesions which can lead to severe destruction of mucous membranes of the nose, mouth, and throat cavities. It is caused mainly by *L. braziliensis* and *L. panamensis*. The cutaneous forms of leishmaniasis are the most common and characterized by the presence of skin ulcers; they represent about 90% of all new cases [5]. Up to 20 species can cause cutaneous leishmaniasis [2]. Unfortunately, treatment options for leishmaniasis are very limited and drug resistance is a big problem [6,7].

**Figure 1:**
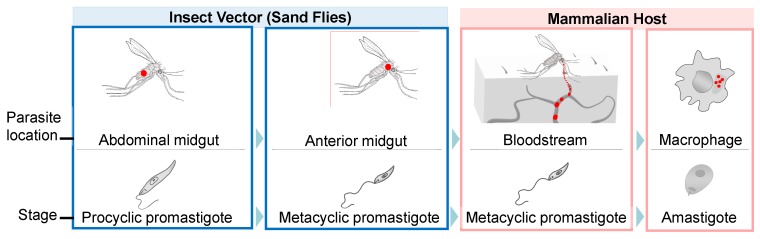
Major stages in *Leishmania* parasite differentiation. During the *Leishmania* life cycle, the parasites transit from phlebotomine sand fly vectors to mammalian vertebrate hosts. In the invertebrate host, *Leishmania* spp. adopts two different stages. The procyclic promastigotes proliferate in the insect abdominal midgut, progressively becoming metacyclic promastigotes migrating toward the anterior midgut. Next, the parasites are transmitted to mammalian hosts during a blood meal. Once in the vertebrate host, the parasites enter phagocytes and transform into intracellular amastigotes. The amastigotes can survive and proliferate inside the phagolysosome, eventually destroying the cell host, infecting new mammalian cells, and restarting an infection round during a new insect bite. Red dots indicate location of parasites.

During their life cycle, these vector-borne protozoans alternate between flagellated promastigotes, which live in extracellular form in the midgut of sand flies, and amastigotes, which reside in the phagolysosomal compartment of mammalian macrophages (Figure 1). Control of leishmaniasis is hampered by the lack of a safe vaccine, limited choice of drugs, their high toxicity, and the emergence of drug resistant strains [8,9].

*Leishmania* parasites possess unique molecular features, such as polycistronic transcription and, as a result, a lack of transcriptional control [10,11]. Long polycistronic transcripts coding for functionally unrelated proteins are produced by RNA Polymerase II. Polycistronic RNA are processed by 5′ trans-splicing of a capped spliced leader sequence and 3′ polyadenylation to generate mature mRNAs [12]. Therefore, the absence of transcriptional control makes *Leishmania* a good model to study post-transcriptional regulation, including mRNA translation representing the major mechanism of gene expression in *Leishmania* species.

When the parasite switches from an insect host to a warm blooded mammalian, it is exposed to a number of stresses including temperature increase, lower pH, and change in nutrients. Environments including temperature, pH, and nutrition conditions play a big role in gene expression regulation [13]; however, it is poorly understood what molecular players are involved in the regulation of translation during environmental stresses and change of hosts. It has been shown recently, based on an example of another protozoan, *Trypanosoma brucei*, that exposure to the stresses is essential not only for the survival of the parasite, but also for its differentiation [14]. Control of gene expression in *Leishmania* species is mostly achieved during translation and by regulating mRNA stability. It is known that translation is globally repressed during heat shock; however, some mRNAs escape translational repression and their translation is enhanced in trypanosomatids, including *Leishmania* species [14,15,16]. Translation of mRNAs encoding for proteins involved in stress response is crucial for the parasite’s ability to cope with stress and its survival. The same players can be essential for the regulation of parasite differentiation when the parasite switches from a sand fly to a mammalian host where it encounters change in temperature, pH, and nutrition [17]. However, it is not well understood how stress-induced mRNAs escape the global translational repression during the heat stress and transmission to mammalian host.

Since *Leishmania* spp. lack transcriptional control, they evolved to have a variety of RNA-binding proteins (RBPs) to control gene expression post-transcriptionally. RBPs promote the differentiation of the parasites and support their survival in invertebrate and vertebrate hosts, where they encounter such a drastically different environment. This fine tuning of gene expression regulation during the life cycle of the parasite is achieved by modulating the mRNA level stability and transcript involvement in the translation.

In this review, we focus on the role of translational control and RBPs in the regulation of the parasite’s life cycle and ability to survive and thrive during transmission to a different host. Special emphasis is given to the role of translational reprogramming in the development of drug resistance. Uncovering of molecular mechanisms of translational control used by *Leishmania* parasites to flourish in different hosts, such as sand flies and mammals, and deciphering the role of translational reprogramming in drug resistance help to identify new pharmacological targets and develop novel treatments in the future.

## 2. Translational Control during *Leishmania* Differentiation

*Leishmania* protozoan parasites have a complex parasitic life cycle, alternating between a sand fly vector and a mammalian host. They exist as highly motile promastigotes containing flagella (in insects) and as amastigotes with very short flagella (in mammals). Flagellated promastigotes live free in the midgut of the insect and undergo a dramatic transformation in the mammalian host, where they become amastigotes living inside of macrophages.

**Figure 2:**
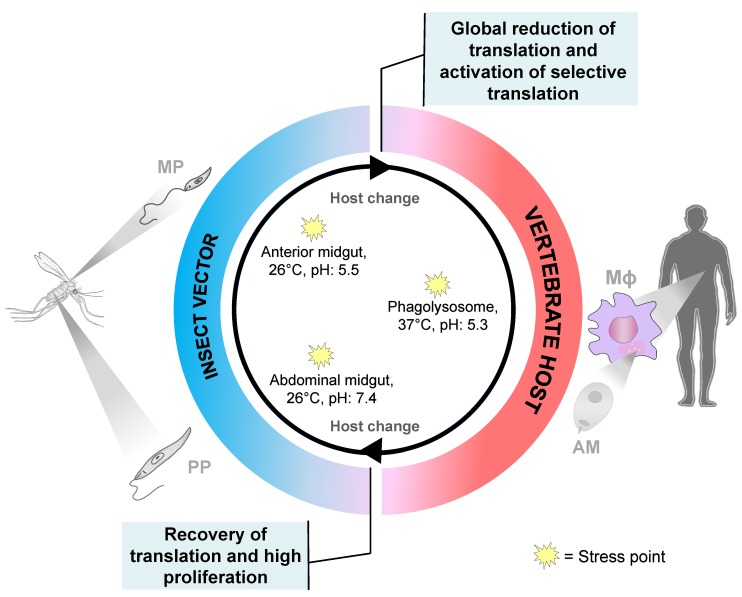
Regulation of translation at different stages of life cycle in *Leishmania* parasites. *Leishmania* parasites undergo dramatic changes in translation during host change. During transition from insect vector to vertebrate host, the changes in temperature, pH, and nutrition promote general translational repression via eIF2α phosphorylation. At the same time selective translation is upregulated in amastigotes, to ensure parasite survival and adaptation in macrophages. In the sand fly, parasites experience a substantial drop in temperature and change in pH and nutrition. It promotes substantial increase in translation and amastigote differentiation to highly proliferative promastigotes. MΦ, macrophage; PP, procyclic promastigotes; MP, metacyclic promastigotes; AM, amastigotes.

*Leishmania* parasites have several steps in the process of their differentiation (Figure 2). *Leishmania* begins its life cycle in the midgut of a sand fly upon infection with the blood meal containing amastigotes. The amastigotes are transformed into proliferative non-infective procyclic promastigote forms when they reach the abdominal midgut. Later, promastigotes undergo morphological and biochemical changes in the process of metacyclogenesis in the invertebrate host and become highly infective motile metacyclic promastigotes. They are characterized by a substantial decrease in RNA, protein, and lipid turnover [18]. Nutritional stress, such as depletion of purines, promotes the differentiation of parasites into virulent metacyclic forms [19,20]. This is characterized by reduced translation rates and appearance of stress granules storing stalled ribosomes. Assembly of stress granules is very important for the parasite survival during nutritional stress experienced in the sand fly. *Leishmania amazonensis* initiation factor LeishIF4E-3, a cap-binding protein paralog, has been found in the composition of nutritional stress granules, upon starvation in vitro [21,22]. It relocates from the cytoplasm to the stress granules in phosphorylated form [20]. Deletion of one allele of *LeishIF4E-3* leads to decline in protein synthesis, inability of parasites to differentiate in the absence of purines, and impaired infectivity [23]. During mammalian host infection, metacyclic promastigotes get engulfed by macrophages, where they transform into amastigotes and multiply. Finally, the sand fly becomes infected during the blood meal on the infected host, completing the life cycle of the parasite.

*Leishmania* parasites have evolved to remodel their cellular architecture and physiology in order to survive in two different hosts with such dramatically different environment. When the parasite is transmitted to a mammalian host, it experiences a big temperature increase, acidic pH, and nutritional stresses, promoting dynamic alterations in gene expression to adapt to the new environment. Due to the lack of transcriptional regulation of gene expression, translational control plays a major role in adaptive responses during the promastigote to amastigote differentiation (Figure 2). All of the above stresses are crucial to drive the promastigote to amastigote differentiation, with the temperature shift being the major driver [24,25]. It has been found recently that mimicking these conditions is sufficient to trigger the differentiation in *L. infantum* in vitro, leading to the decrease in global mRNA translation [17]. This decrease during amastigote differentiation correlates with the phosphorylation of the alpha-subunit of eukaryotic initiation factor 2 (eIF2α). In eukaryotes, phosphorylation of eIF2α is one of the major stress response pathways leading to the global reduction in translation [26,27,28]. Interestingly, while global translation has been shown to be reduced during amastigote differentiation, translation of amastigote specific transcripts is selectively up-regulated [17]. Translating ribosomes and polysomes can be fractionated to determine the efficiency of translation and mRNA engagement, and mRNA association with heavier fractions of polysomes indicates its efficient translation [29]. It was demonstrated that the A2 amastigote-specific transcript is shifted to heavy polysomes in *L. infantum* axenic cultures and leads to its increased translation, as evident from the increase at the protein level [17]. Recent work on *Trypanosoma brucei* procyclic forms has found that many heat-induced mRNAs are increased during the differentiation to mammalian-infective forms [14]. Heat shock caused global inhibition of translation and sequestration of mRNAs in stress granules. However, heat-induced mRNAs were spared from translational repression and sequestration to stress granules, through binding to the ZC3H11 protein. ZC3H11 is an RNA binding protein which stabilizes selective mRNAs during the heat shock and allows their translation. It is possible that a similar mechanism exists in *Leishmania* species to support selective translation during the differentiation in the mammalian host when the parasite encounters a temperature increase and other stresses; however, this question needs to be addressed in the future. Thus, the global translational repression is observed while the selective translation of amastigote specific transcripts is upregulated suggesting a global reprogramming of translation to accommodate a successful parasite survival in the mammalian host; however, many details of this reprogramming remain unknown.

Initiation of translation may play a big role in translational reprogramming when parasites enter a different host. Initiation of translation takes place through the binding of cap-binding protein eIF4E to the 5′ cap structure, along with its binding partner eIF4G which serves as a scaffold protein [20]. Then, a large ribosome subunit is recruited when the initiation complex reaches the first AUG [27]. Interestingly, the trypanosomatid genomes including *Leishmania* species have a large number of cap-binding complexes including six paralogs of eIF4E and five paralogs for eIF4G scaffold protein [30,31,32,33,34,35]. The presence of many paralogs with different cap-binding activities and expression profiles during the life cycle of the parasite suggest that each paralog has evolved to perform a different biological function and a very complex regulation of gene expression, during the initiation of translation to support parasite survival under constantly changing environments. However, the precise role of these proteins in *Leishmania* parasites remains unclear and requires further investigation.

## 3. RNA Binding Proteins and Their Role in Regulation of Translation and Differentiation of Parasites

**Figure 3:**
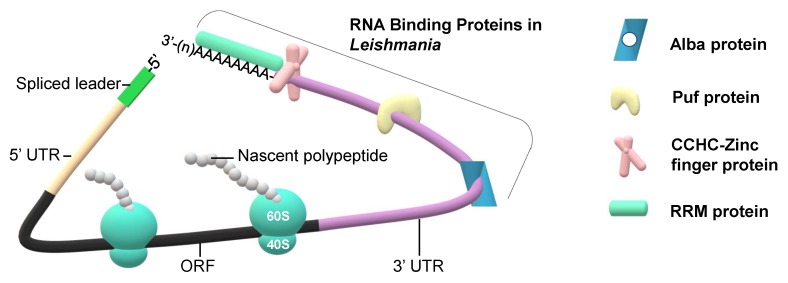
Schematic representation of RNA binding proteins (RBPs)’ interaction with mRNA during translation in *Leishmania* parasites. *Leishmania* RBPs are grouped in four main classes, Alba (acetylation lowers binding affinity) proteins, Puf (Pumilio and Fem-3 binding factor) proteins, CCCH zinc finger proteins, and RNA-recognition motif (RRM) proteins. RRMs include proteins binding mRNAs in different part of 3′ untranslated region (3′UTR) and poly(A). Poly(A)-binding protein (PABP) is shown as an example of the RRM proteins. RBPs modulate selective translation by mRNA stability, mRNA decay, polysome association, or mRNA storage process.

Gene expression is predominantly controlled at the translational level in *Leishmania* parasites, therefore, regulation of mRNA stability/translatability by RNA binding proteins (RBPs) is essential for the fine tuning of translation during different stages of the life cycle of the parasite. Despite the lack of transcriptional control in *Leishmania*, the regulation at the mRNA levels still exists and is accomplished by post-transcriptional mechanisms. Therefore, RBPs play a big role in the regulation of mRNA levels achieved by the modulation of mRNA stability/degradation. Trypanosomatids including *Leishmania* have large number of RBPs due to their primary role in the regulation of expression in the absence of transcriptional control, however, the vast majority is lacking known orthologues in other eukaryotes [36]. Searching of the *Leishmania donovani* genome for the presence of RNA binding proteins identified 67 proteins in total, and analysis of mRNA interactome revealed 79 RBPs [37]. There are several major classes of RBPs each with distinct functions in post-transcriptional gene expression regulation: RNA-recognition motif (RRM) proteins, CCCH zinc-finger domain proteins, Puf (Pumilio and Fem-3 binding factor) domain proteins, and Alba (acetylation lowers binding affinity) proteins (Figure 3).

*Leishmania major* has 78 RRM proteins [10], while *Trypanosoma brucei* has about 70 RRM proteins and half of them are essential in at least one stage of life cycle [38,39]. RRM proteins play an important role in the regulation of mRNA stability and the fine tuning of translation promoting adaptation and survival during environmental challenges encountered by the parasite at different stages of the life cycle. RRM proteins contain at least one RRM domain consisting of a 90 amino acid-long module, one of the most commonly existing domains in nature. RRM proteins may interact with regulatory sequence elements in 3′UTRs of mRNAs to control their stability and translatability under different environmental conditions. This regulation is especially important for proper differentiation of the protozoan parasites. While little is known about RRM proteins in *Leishmania*, many studies have been performed in *Trypanosoma*. Several RBPs containing an RRM domain have been found to have a profound life-cycle stage-specific effect on the global regulation of the parasite transcriptome and its development [40]. RBP10 expression is upregulated in bloodstream-form trypanosomes and it modulates mRNAs typically found in bloodstream-form parasites [41,42]. Conversely, *Trypanosoma brucei* RBP6 plays a crucial role in the insect stage and its forced overexpression leads to the progression of metacyclogenesis, activation of variant surface glycoprotein genes expression, and infectivity [43]. Thus, both RBP6 and RBP10 are important developmental triggers supporting the critical role of RBPs in the post-transcriptional regulation of gene expression. It is possible that RBP6 and RBP10 homologs exist in *Leishmania* and perform similar roles.

Poly(A)-binding proteins (PABPs) belong to RRM proteins and contain a single RRM domain. Poly(A)-binding proteins in eukaryotes play a role in polyadenylation of the transcripts in the nucleus, mRNA circularization via binding to translation initiation factors, and cause the enhancement of translational initiation [44,45,46]. *Leishmania* protozoans have three PABPs, but only two of them PABP1 and PABP2 are conserved in trypanosomatids [47,48,49,50]. All three are highly expressed and can bind poly(A), however, only PABP2 and PABP3 interact with each other and migrate to the nucleus upon inhibition of transcription. PABP1 can be associated with the eIF4E4/eIF4G3 complex via direct binding to eIF4E4 to regulate the initiation of translation [30]. PABP1 is expressed at a constant level during differentiation in *Leishmania infantum*, however, it is hyperphosphorylated during active translation and displays a stronger association with polysomes in logarithmically grown cells [47]. Both PABP1 and PABP2 can stimulate translation and can be associated with polysomes in *Trypanosoma brucei*, however, they exhibit differences in the association with polysomes and intracellular localization [49,51,52]. PABPs also play an important role in mRNA decay and can be localized to distinct stress granules in *Leishmania*. LeishPABP2, but not LeishPABP1, is found in the composition of nutritional stress granules, but it still remains unclear what role it may play in the differentiation of the parasite [20].

The CCCH (Cys_3_His Zinc finger) proteins are characterized by the presence of defined zinc-finger motif with preference to bind AU-rich elements in RNA [40]. *Trypanosoma* ZFP1 and ZFP2 proteins belong to the CCCH family and both of them are important for the differentiation from bloodstream to procyclic forms [40,53,54]. While ZC3H20 is needed for procyclic forms, ZC3H11 is essential in bloodstream parasites [55,56]. ZC3H11 is suggested to act as a platform in the recruitment of PABP to 3′UTR and to regulate mRNA stability and translation [57]. ZC3H11 protects a subset of mRNAs from translational repression during the heat shock, and many of heat-induced mRNAs are also elevated during differentiation to mammalian-infective forms [14]. Bioinformatic analysis of the TriTryp genome database identified that *Trypanosoma brucei* has 48 CCCH proteins, while *Leishmania major* carries 54 proteins with 8 of them being unique; however, the role of CCCH proteins in the regulation of *Leishmania* parasite differentiation has not been addressed yet [58,59].

Puf proteins are known to be involved in the regulation of mRNA localization, stability, and translation, through binding to the sequence motif in the 3′UTR of specific mRNAs [60,61,62]. Puf proteins contain an RNA binding domain consisting of several imperfect amino acid repeats called Puf repeats. Puf proteins promote translational repression and mRNA degradation via interactions with cis-elements in the 3′UTR of specific mRNAs [63,64,65,66]. Both *Leishmania* and *Trypanosoma* species contain up to 10 different Puf proteins [67,68,69,70,71]. Puf6 protein regulates selective transcript levels during the life cycle of the parasite via mRNA degradation [72,73]. Puf proteins could be found together with LeishIF4E-3 in starvation-induced stress granules in *Leishmania* [20]. However, in general, there is very little known about the biological functions of Puf proteins in *Leishmania*.

Other proteins interacting with 3′UTRs are Alba proteins [74]. They are abundant mRNA-binding proteins regulating translation in trypanosomatids and can be found in association with polysomes and translating mRNAs [75]. The expression of Alba proteins is stage-regulated in *Trypanosoma* and contributes to the differentiation of the parasites during their development in the tsetse fly [76]. While *Trypanosoma* has four proteins with Alba domain, *Leishmania infantum* genome has only two Alba proteins [39,77]. LiAlba3 protein can bind the delta-amastin 3′UTR and regulate amastin mRNA stability in *Leishmania infantum* during the amastigote stage [74]. Both Alba proteins were found associated with ribosomal subunits in *Leishmania*, in contrast with *Trypanosoma*, where Alba proteins are observed in polysomes; however, it is unclear how it contributes to the differences in function [78].

Thus, *Leishmania* parasites have a great variety of RNA binding proteins (RBPs) playing especially important role in the regulation of protein translation through controlling mRNA stability/translatability, and providing fine tuning of protein translation during differentiation of the parasite in the absence of transcriptional control. While *Leishmania* life cycle stages are well studied, we are just at the beginning of understanding what molecular mechanisms of translational control operate at the different stages of the parasite’s life cycle, and how different RBPs contribute to it.

## 4. Drug Resistance and Translation

Current treatment of leishmaniasis is obstructed by drug toxicity, high cost, and treatment failures caused by drug resistance [6]. Many studies are focused on drug resistance now, since it is a major public health problem worldwide associated with the disease incidence, mortality, and health care cost. Traditionally, pentavalent antimonials (Sb^V^) have been used as the primary therapeutic treatment option against leishmaniasis [79]. However, drug resistance has been widely shown to reduce the therapeutic efficacy of Sb^V^ [7]. In certain regions of India and Nepal, therapeutic failure of Sb^V^ has reached 65% leading to discontinuation of the treatment [7]. Rising rates of Sb^V^ drug resistance have been reported in Latin America as well [80,81]. Unfortunately, *Leishmania* parasites have also shown the ability to develop drug resistance against other therapeutic alternatives including amphotericin B, miltefosine, pentamidine, and paramomycin [82,83,84]. Furthermore, cross-resistance phenomena have been widely documented, suggesting a high capacity of *Leishmania* to adapt to different stressors and drugs [85,86,87,88,89,90]. In the absence of both an effective vaccine and a stable therapy for leishmaniasis treatment, there is an urgent need to understand the drug resistance mechanisms adopted by these parasites.

**Figure 4:**
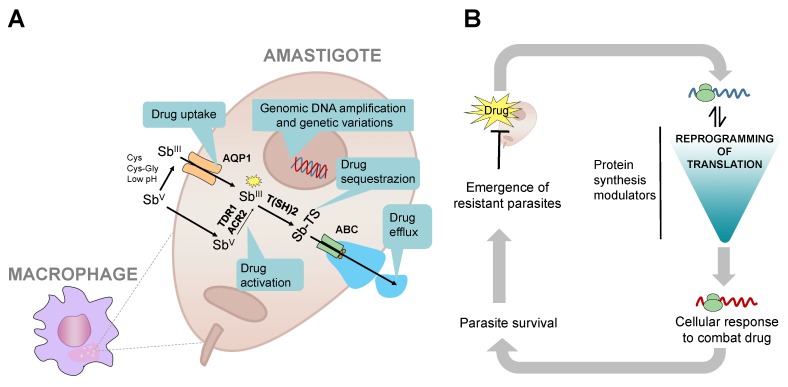
A schematic representation of Sb^V^ resistance in *Leishmania* parasites. (**A**) Genomic amplifications, mutations, and genetic variations contribute to Sb^V^ resistance development. Several mechanisms can contribute to the drug resistance. Down-regulation of AQP1 blocks the drug uptake. Down-regulation of reductase enzymes prevents drug activation from Sb^V^ to Sb^III^. Increase in drug sequestration can be achieved through action of thiol-metal conjugates (Sb^III^-TS). Changes in drug efflux across ABC transporters and vesicles released by exocytosis also contribute to Sb^V^ resistance phenotypes. (**B**) Scheme representing coordinated reprogramming of translational activity during the development of resistance to Sb^V^ and other drugs. Once the drug pressure is initiated, the parasites sense the stress and initiate response. The translation is reprogrammed by modulation of proteins such as CDPK1 to combat the toxic action of drug. Some translational regulators could participate as a switch inducing or inhibiting the protein synthesis, prioritizing the production of essential proteins. These proteins could be involved in mRNA and protein stability, lipid metabolism, stress response and drug depuration/inactivation, promoting the parasite survival and drug resistance. Thiol-dependent reductase I (TDR1), As/Sb Reductase (ACR2), trypanothione (T(SH)2), aquaglyceroporin (AQP1), ATP-binding cassette (ABC), cysteine (Cys), cystein–glycine (Cys-Gly), and antimony-thiol conjugate (Sb-TS) are shown.

The drug resistance studies in *Leishmania* have mainly focused on Sb^V^, since it is the main drug currently used for treatment. The studies suggest that *Leishmania* uses at least four main mechanisms to counteract Sb^V^ drug: (a) reduction in drug uptake; (b) prevention of drug activation; (c) drug sequestration; and (d) increase in the drug efflux (Figure 4A).

A reduction in the drug uptake can be achieved by downregulation of membrane proteins as aquaglyceroporin (AQP1) [91]. The drug activation might be blocked by negative regulation of specific reductase enzymes, protecting parasites by preventing the reduction from Sb^V^ (prodrug) to trivalent antimony (Sb^III^, active drug form). The Sb^V^ action can be inactivated by the production of metal-thiol conjugates, leading to the drug sequestration. Finally, the overexpression of ABC transporters increase the drug efflux of metal-thiol conjugates across vesicles that fuse with the plasma membrane during exocytosis [92].

Different approaches have been used to understand Sb^V^ resistance in *Leishmania*. Studies at the genomic level have shown that single nucleotide polymorphism and genomic amplification including copy number variation [93], chromosomal somy [94], and intrachromosomal [95] and extrachromosomal amplification contribute to Sb^V^ resistance. However, changes at the genomic level cannot explain all the possible drivers of resistance mechanisms. Interestingly, 1006 transcripts are differentially expressed during the experimental development of resistance to trivalent antimony in *L. donovani* [96]. Deep RNA-sequencing studies have further shown that Sb^V^ sensitive and resistant strains exhibit quite different mRNA profiles, suggesting global changes in gene expression [93,97] In the Sb^V^ resistance strains, the next pathways are enriched: energy metabolism (glycolysis and TCA cycle), phosphate ion transport, metabolism of fatty acids, biosynthesis pathway of trypanothione, and stress response [96,97,98]. Taking into account that *Leishmania* does not have transcriptional control and that mRNA stability is tightly connected with their translation, the global changes in gene expression profile support the hypothesis that the translational reprogramming could orchestrate the drug resistance development in *Leishmania* parasites (Figure 4B). Recently, it has been discovered that the essential protein, calcium-dependent protein kinase 1 (CDPK1) is able to modulate the translation efficiency, and mutations in this protein contribute to paramomycin and antimony resistance [9]. CDPK1 phosphorylates the ribosomal protein L23a, and it also interacts with the ribosomal protein L28 and ARM56 [9]. Interestingly, these proteins are also related to drug-resistance; L23a over-expression confers resistance not only to Sb^V^ but also to miltefosine and paramomycin, suggesting a general system for drugs-stress response [99]. Furthermore, the putative 60 S ribosomal protein L28 is highly expressed in Sb^V^ resistance strains [94] and ARM56 has been proposed as a clinical Sb^V^ resistance marker [100]. It remains to examine if the phosphorylation pattern and the interactome of CDPK1 has a central role for the translational reprogramming during drug response.

It is well documented that reprogramming of mRNA translation plays a key role in drug resistance during cancer treatment and targeting specific elements of the translation machinery is emerging as an innovative strategy for cancer therapy [101,102,103,104,105]. Although the drug resistance in *Leishmania* has been studied for the past 20 years, the role of the translational regulation has remained essentially neglected. The studies discussed here suggest that under drug pressure, *Leishmania* should activate a coordinated reprogramming of translation, modulating the selective mRNA translation and activating pathways to combat the drug (Figure 4B). This process may be highly specialized in trypanosomatid organisms that lack transcriptional control. While the data implicate the importance of translational control, the uncovering of how parasites reprogram their translation to withstand the presence of drug and develop resistance requires future studies.

## 5. Conclusions and Perspectives

The goal of this review is to provide the current progress achieved in studies on translational control in *Leishmania* parasites. Translational control is the major mechanism promoting the transformation of promastigotes (sand fly stage) into amastigotes (mammalian stage) achieved by decreased global translation via eIF2α phosphorylation. Initiation of translation factors and a huge variety of RNA binding proteins are all important for the differentiation, however, precise biological functions have been characterized only for a few of them. Further investigation of the role of different translational regulators and RBPs is necessary for a better understanding of the biology of the digenic parasite.

Drug resistance is one of the major problems in the treatment of the leishmaniasis and circumventing the problem of drug resistance is critical for successful treatment in the future. In order to combat this disease effectively, a better understanding of the fine mechanisms of drug resistance development is essential. While changes at genomic levels that contribute to the development of drug resistance are very well studied, the role of translational reprogramming remains largely unexplored. The understanding of the machinery responsible for global translational reprogramming during drug resistance development will be very valuable in developing new highly effective therapeutic alternatives for leishmaniasis treatment.

## Abbreviations

eIF2α	alpha-subunit of eukaryotic initiation factor 2
RBPs	RNA binding proteins
Puf	Pumilio and Fem-3 binding factor
Alba	acetylation lowers binding affinity
RRM	RNA-recognition motif
PABP	Poly(A)-binding protein
CCCH	Cys_3_His Zinc finger
ZFP1	zinc finger protein 1
ZFP2	zinc finger protein 2
UTR	untranslated region
CDPK1	calcium-dependent protein kinase 1
TCA	tricarboxylic acid cycle
